# COVID-19 and Civil Society in Southeast Asia: Beyond Shrinking Civic Space

**DOI:** 10.1007/s11266-022-00496-1

**Published:** 2022-04-29

**Authors:** Jasmin Lorch, Janjira Sombatpoonsiri

**Affiliations:** 1 German Institute for Global and Area Studies (GIGA) and Willy Brandt School of Public Policy, University of Erfurt, Erfurt, Germany; 2 German Institute for Global and Area Studies (GIGA) and Institute of Asian Studies, Chulalongkorn University, Bangkok, Thailand

**Keywords:** COVID-19, Shrinking space, Civic space, Civil society, Southeast Asia

## Abstract

In this article we challenge the conventional wisdom that COVID-19 and related legal restrictions invariably reinforce a global trend of shrinking civic space. We argue that the legal guarantee (or restriction) of civil society rights is not the sole factor configuring civic space. Instead, we reconceptualize civic space by broadening its determinants to also include needs-induced space and civil society activism. Investigating five countries with flawed democracic or competitive autocracic regimes in Southeast Asia, we propose a three-pronged mechanism of how these determinants interact in the context of COVID-19. First, legal restrictions on civil society rights intertwine with the space created by health and economic needs to create new opportunities for civil society activism. Second, these new opportunity structures lead to the cross-fertilization between service delivery and advocacy activism by civil society. Third, this new trajectory of civil society activism works to sustain civic space.

## Introduction

One and a half years into the COVID-19 pandemic, discussions about its impact on civic space are ongoing in academic and policy circles. The most common assumption is that lockdowns and other restrictive measures to curb the spread of the coronavirus erode what CIVICUS (2021) terms as the three key “civil society rights” of association, peaceful assembly, and free expression, thereby reinforcing a preexisting global trend of shrinking civic spaces (e.g., CIVICUS, 2020, V-Dem, 2020; on long term trends, see Carothers & Brechenmacher, 2014; Dupuy et al., 2016; Poppe & Wolff, 2017). This is particularly so as incumbents in many regimes with democratic deficits use the pandemic to strengthen their rule, for instance by invoking draconian laws aimed at stifling civil society (Bethke & Wolff, 2020; ICNL, 2021; Smith & Cheeseman, 2020). For instance, in addition to limiting interpersonal contacts, many regimes have restricted access to information and freedom of expression, detained activists, and relied on the military to enforce COVID-19-related measures (Bethke & Wolff, 2020; CIVICUS, 2020).

In contrast, some imply that the pandemic also has some positive impacts on civic space. Research by the Carnegie Endowment for International Peace finds that civil society worldwide has shown remarkable “dynamism despite disruption” (Brechenmacher et al., 2020) and that the pandemic has acted as a “catalyst for global civil society” (Youngs, 2020). Specifically, formal CSOs, informal community-based organizations (CBOs), and social movements have actively responded to the health crisis and its economic side effects (Asia Foundation, 2020; EESC, 2021), filling gaps left by states (Shapovalova, 2020).

This view is in line with existing studies suggesting that structural constraints are not necessarily always effective in quelling civil society activism. The nascent literature on civil society pushback against “NGO laws” that limit CSOs’ access to foreign funding and label them as foreign agents shows that civil society can sometimes avert legal onslaughts (Berger-Kern et al., 2021). Similarly, the autocratic use of laws to persecute civil society can backfire by fueling protests (Sombatpoonsiri, 2021). Research on weak states shows that the failure of state institutions to meet socioeconomic needs can open spaces for civil society in the welfare sector and promote the emergence of CSOs in fields such as health and education (Lorch, 2017, 38–40). Under these circumstances, civic space may not necessarily shrink, but rather grow or change (Alscher et al., 2017; Anheier et al., 2019; Toepler et al., 2020).

Underlying the diverging assessments regarding the negative or positive impact of COVID-19 on civic space are different understandings as to what determines civic space. Proponents of the assumption that civic spaces are further shrinking consider the pandemic as a structural constraint, focusing predominantly on how COVID-19 related legal measures further curtail civil society’s rights to the freedoms of assembly, association and speech and obstruct CSOs’ operations. By contrast, research implying that civic spaces have persisted or expanded focuses on new opportunities COVID-19 offers for civil society activism.

We build on these existing works to conceptualize civic space as being shaped by three interrelated determinants: first, the legal guarantee or restriction of civil society rights that affect CSOs’ operations; second, socioeconomic needs that configure needs-induced space; and, third, civil society activism. Furthermore, we propose that these determinants interact in three main ways in the context of COVID-19. First, legal measures that limit civil society rights intertwine with needs-induced space to create new opportunities for civil society activism. Second, these new opportunity structures lead to the cross-fertilization between service delivery and advocacy activism by civil society. Third, this crossover of civil society activism works to sustain civic space.

To investigate this three-pronged mechanism, we focus on five countries with flawed democratic or competitive autocratic regimes in Southeast Asia–Malaysia, Indonesia, Thailand, the Philippines, and Myanmar—whose governments have passed new laws to undermine civil society rights but have simultaneously depended on CSOs to deliver welfare services during the pandemic. We first introduce our argument and explain our research strategy. We then sketch political developments in the five countries before COVID-19. Subsequently, we show how the interplay of legal restrictive measures, needs-induced space, and civil society activism has sustained civic space during the pandemic. We conclude by discussing the implications of our findings for future research and for civil society in Southeast Asia.

### Rethinking Civic Space: Needs as Opportunities, Cross-fertilization of Activism, and Sustained Space

We consider civic space as the sphere in which civil society can operate de facto (Alagappa, 2004, 50–52; Alscher et al., 2017, 11). Drawing on Kaldor (2003, 44–47), civil society is a set of nongovernmental institutions that are self-organizing, not-for-profit, and usually independent of the state. Civil society comprises diverse actors, including formal CSOs, informal CBOs, and social movements, which may not necessarily be democratic (Alagappa, 2004). Similarly, CSOs’ mandates can be heterogeneous, including advocacy and service provision (Toepler & Anheier, 2020, 8).

We bring the literature on shrinking space together with the wider scholarship on civil society and social movements to derive three determinants that shape civic space. The first is legal regulations that guarantee or restrict the civil society rights to associate, assemble, and freely express views (CIVICUS, 2021), or, in other words, *rights-based space*. Rights-based space shrinks when governments rely on draconian laws, from so-called NGO laws to anti-terror and anti-fake news laws, to brand critical CSOs as foreign agents and security threats, or justify crackdowns (Carothers & Brechenmacher, 2014, 9–10; Dupuy et al., 2016). COVID-related legal measures can reduce rights-based space by enhancing governments’ exercise of executive powers and facilitating infringements on civil society rights (Kuehn et al., 2021). While important new literature rightly points to the need to study regulatory regimes governing CSOs in their entirety (DeMattee, 2018), such an approach is beyond our scope. Instead, we focus on COVID-19-related legal measures, contending that whether such measures effectively diminish civic space also depends on two other determinants.

The second determinant is the existence (or absence) of socioeconomic *needs-induced space*. By socioeconomic needs, we imply basic requirements for human beings to achieve a decent life, including food and shelter, education, healthcare facilities, and employment (Chiappero-Martinetti, 2014). When states fail to fulfill these requirements, needs-induced space for civil society emerges. Governments, including autocratic ones, make “cost–benefit calculations” (Berger-Kern et al., 2021, 85) and may hence allow CSOs to bridge gaps in fields such as health, food, and education to prevent aggrieved citizens from engaging in disruptive actions that destabilize their rule (Liverani, 2008; Lorch, 2006). If regimes rely heavily on CSO services, this sometimes allows civil society to expand its scope for political action (Liverani, 2008; Lorch, 2006). In the context of COVID-19, needs-induced space for civil society emerges when government responses, such as lockdowns, impact the livelihoods of vulnerable groups, such as informal-sector workers (United Nations, 2020, 1). Hence, while restrictive legal measures may shrink rights-based space (determinant 1), their socioeconomic side effects potentially foster needs-induced space.

The third determinant is civil society activism. Inline with the research on civil society, we focus on two types: service provision and advocacy (Edwards & Hulme, 1996). By service delivery, we imply the provision of social services to address socioeconomic needs. By advocacy, we imply CSOs trying to convince policy makers or the general public to support their agenda. The methods used can be institutional (e.g., formal dialogue with government representatives) or extra-institutional (e.g., street protest and strikes) and aim at furnishing CSOs with influence to realize their demands (Cinalli & Giugni, 2014).

We expect that these three determinants interact in three main ways. First, based on Social Movement Studies’ insights on political opportunity structure, contexts such as repression and economic needs can create opportunities for civil society mobilization by undermining governments’ legitimacy, fomenting public support for movements that voice popular grievances, and galvanizing collective actions (Tarrow & Tilly, 2009). We expect the nexus of COVID-19-related legal measures and needs-induced space to similarly produce political opportunities for civil society activism. Hence, our first proposition is that,

(i) Restrictive legal measures and the emergence of needs-induced space intertwine to create political opportunities for civil society activism.

Second, the civil society activism that has emerged during COVID-19 reflects the cross-fertilization between service-delivery and advocacy. Legal restrictions such as lockdowns have often driven contentious activism, because they have inflicted socioeconomic predicaments (Pinkney & Rivers, 2020). In addition, typical advocacy CSOs, such as human rights and pro-democracy groups, have included welfare demands into their agendas and provided services to vulnerable groups, while service-providing CSOs have pressured governments to support populations in need and adopt new models of economic development (Carnegie Civic Research Network, 2021, 4–5; Pleyers, 2020). Thus our second proposition is that,

(ii) The interaction between restrictive legal measures and needs-induced space leads to the cross-fertilization of civil society’s service delivery and advocacy activism.

We assume that the cross-fertilization of service delivery and advocacy activism sustains civic space in several ways. First, due to governments’ dependence on their contributions, service-delivering CSOs may gain some leverage vis-à-vis their governments, which they can utilize to advance advocacies related to socioeconomic needs (Cinalli & Giugni, 2014). Second, engaging in service provision may allow advocacy CSOs, such as human rights organizations, to continue working despite legal restrictions. Once involved in social work, such CSOs may promote “rights-based approaches” and strengthen the “advocacy dimension” of service provision (Clayton et al., 2000, 21), thereby politicizing formerly apolitical spaces in social service delivery. Third, COVID-19 reorients CSOs’ agendas around socioeconomic narratives, potentially allowing them to “lobby against civic space restrictions” (Berger-Kern et al., 2021, 84) by framing government failures to tackle COVID-19 as the result of longer-standing structures of political and socioeconomic inequality and exclusion. Thus, our third proposition is that,

(iii) The cross-fertilization of service delivery and advocacy activism works to sustain civic space.

Our investigation of five Southeast Asian countries with flawed democratic or competitive autocratic regimes substantiates these three propositions.

### Research Strategy

We study Indonesia, Malaysia, Thailand, the Philippines, and Myanmar, which are religiously, ethnically and culturally diverse but share four conditions relevant to our three propositions. First, they have a population of more than 1.5 million. We consider this as an appropriate ceiling for capturing countries with sufficiently available information (see below) and with a number of inhabitants that allows for cross-country comparisons. Brunei and Timor Leste, which have fewer inhabitants, are excluded. Second, in all five countries, the incumbent regimes have attempted to curtail civic space through legal measures. Third, all five are middle-income countries (World Bank, 2020), which has limited the ability of their states and economies to tackle the health and socioeconomic needs caused by COVID-19. We exclude Singapore, because it is a high income country (World Bank, 2020), whose state institutions have generally disposed of the resources necessary to address these needs. And, fourth, all five are either flawed democracies or competitive autocratic regimes that scored above 25 and at least one time ranked as “partly free” on the Freedom House Index between 2018 and 2020 (Freedom House, 2019, 2020, 2021). Flawed democracies are regimes with democratic elections that guarantee civil liberties in principle but display notable democratic deficits due to weak governance (EIU, 2021, 57). Competitive autocracies hold passably free and fair elections but simultaneously create an unlevel playing field and curtail civil liberties to such an extent that alternations of power are severely restricted (Levitsky & Way, 2002). We limit our observation to these regime types, because they guarantee a minimum threshold of civil society rights, making it possible for independent CSOs to exist. Moreover, they provide for an information environment that is free enough to allow us to access empirical evidence about civil society activities. We exclude the closed autocracies of Laos, Vietnam, and Cambodia, which severely restrict the flow of information, making the gathering of information about civil society activities impossible without extended field research.

Our empirical evidence is drawn from three sets of sources, following our propositions regarding the three determinants shaping civic space. First, to investigate COVID-19-related legal measures impacting civic space, we rely on the International Center for Not-for-Profit Law’s (ICNL) COVID-19 Civic Freedom Tracker as the most comprehensive and well-established database to trace COVID-19-related legal measures. We focus on the timeframe from April 2020 to February 2021, as most governments enacted their main COVID-related legal measures during this period. We apply two criteria to code altogether 12 types of legal measures. The first criterion focuses on whether a legal measure was enacted by an executive body (as order or decree) or by a legislative body (as law). The former signals the concentration of executive power, which the literature associates with executives’ intimidation of critical civil society (Kuehn et al., 2021). Regarding laws, we code both legislations and regulations—the latter defined as measures that specify the implementation of laws.

For each of the two categories (laws and decrees), we also apply the second criterion that pertains to six characteristics of the individual legal measures. These are health- or security-oriented emergency measures; curfew orders; bans on public gathering conducive to criminalizing public assemblies even if they follow health regulations; control of information; and so-called NGO laws to restrict foreign funding to and registration of NGOs. We do not consider purely health-related emergency measures as autocratic encroachment on rights-based civic space because they do not necessarily provide all-encompassing power to governments to restrict civil society rights. By contrast, the other five types of measures violate freedoms of association, assembly, and expression and allow regimes to criminalize CSO activities (Bethke & Wolff, 2020; Poppe & Wolff, 2017). In particular, security-oriented emergency measures provide the executive with wartime-like power and can lead to militarized public-health management and eroding rights (Passos & Acácio, 2021).

To explore protests as one form of advocacy activism, we draw on the Armed Conflict Location and Event Data Project (ACLED) as the most well-established and fine grained database to capture COVID-19-related protests (ACLED, 2021). We count the number of protest events with over 100 participants according to their drivers: (1) defiance against COVID-19-related legal measures; (2) economic setbacks; (3) the lack of effective government responses to the health crisis and related economic challenges; (4) corruption allegations related to government public-health management; and, (5) gender-related violence during lockdowns. Thereby, protests prompted by drivers (2), (3), (4), and (5) also signal the emergence of needs-induced space and illustrate how socioeconomic needs can act as a political opportunity for civil society activism.

We drew on gray literature, press reports, and expert opinions in order to identify less disruptive forms of civil society advocacy, to examine whether and how civic space changed due to the intensification of socioeconomic needs, and to assess service delivery by CSOs. In identifying the relevant gray literature, we checked the websites of major domestic NGOs and INGOs as well as of the main bilateral, EU and UN development agencies working in the five countries studied. Regarding press reports, we focused on the main national online newspapers available in English. We also relied on keyword search and snowballing techniques. Moreover, in June 2020 we organized a behind-closed-doors, online roundtable with ten members of Asian CSOs, including human rights organizations, development NGOs, and CBOs that operate in Thailand, Myanmar, and the Philippines. We conducted two additional interviews with CSO experts from Myanmar.

### Southeast Asia’s Civic Space: Pre-COVID-19 Trends

Already prior to COVID-19, rights-based civic space in Indonesia, Malaysia, Myanmar, Thailand, and the Philippines had shrunk due to democratic decline (Croissant & Diamond, 2020). In Thailand, the military regime of General Prayut that seized power through the 2014 coup drafted a new constitution that severely limits competition, and the government remains dominated by the military. In Indonesia, the government of Widodo has curbed the power of the Anti-Corruption Agency and increasingly repressed critics (Mietzner, 2021). By early 2020, the country was classified as a “flawed democracy” (Croissant & Diamond, 2020). The Philippines meanwhile was on its way from being a flawed democracy to a (competitive) autocratic regime (see also, Croissant, 2020), as the government of populist president Duterte cracked down on oppositional voices and committed massive human rights violations.

In Malaysia, the reformist government of Pakatan Harapan had to step down in early 2020. It was replaced by a Malay-centric nationalist coalition led by the United Malays National Organisation, which had dominated politics already from 1975 to 2018, turning the country back to a competitive autocracy (Croissant & Lorenz, 2017). In Myanmar, the Aung San Suu Kyi government’s failure to implement far-reaching reforms stalled the country’s democratic progress, making it a competitive autocracy before the February 2021 military coup (Croissant & Diamond, 2020).

Except for Myanmar, most regimes have not been inclined to rely solely on brute force, while using legal and financial crackdowns to censor dissent and drain the resources of CSOs. The five regimes have also imposed bureaucratic hindrances and financial restrictions on CSOs. As of early 2020, Malaysia and Myanmar had laws that restrict the formation of CSOs deemed too contentious, such as human rights or pro-democracy organizations (Sombatpoonsiri, 2020a). Meanwhile, virtually every country in Southeast Asia passed information-related or cyber laws prior to COVID-19, inflicting heavy penalties on alleged violators (Sombatpoonsiri, 2020b). However, continued and enhanced civil society activism in the context of COVID-19 contrasts with these tendencies.

### Civic Space during the Pandemic

#### Legal Restrictions and Needs-Induced Space as Opportunities

Confirming our first proposition, *COVID-19-related legal measures have further curtailed rights-based civic space, but, together with the emergence of needs-induced space, have also created new political opportunities for civil society activism.* COVID-19-related legal measures passed between April 2020 and February 2021 have further shrunk rights*-*based civic space in the five countries studied. Based on our first criterion, we classified the legal measures in terms of executive versus legislative enactment and found that: (1) altogether seven executive orders or decrees were passed by the executive; and (2) altogether two laws were passed by a legislative body, along with two implementing regulations. When viewed in conjunction with their specific contents (our second criterion), we found that:

- Three executive orders or decrees are related to the health emergency, such as Indonesia’s Presidential Decree No. 11 that declares a “Public Health Emergency.” This endows the executive with special powers and restricts movement and public gathering, but does not frame the health emergency as a matter of national security.
- Three executive orders or decrees draw on wartime-like emergency powers, thereby securitizing government responses to COVID-19.
- One law (the Philippine “Bayanihan to Heal As One Act”) also invokes wartime-like emergency responses to the pandemic.
- One executive order (Myanmar’s Order on Movement) enforces specifically curfews.
- One law specifically bans public gathering (Myanmar’s Order No. 37/2020).
- Two regulations (in Indonesia) specify the implementation of social-distancing measures.

**Fig. 1:**
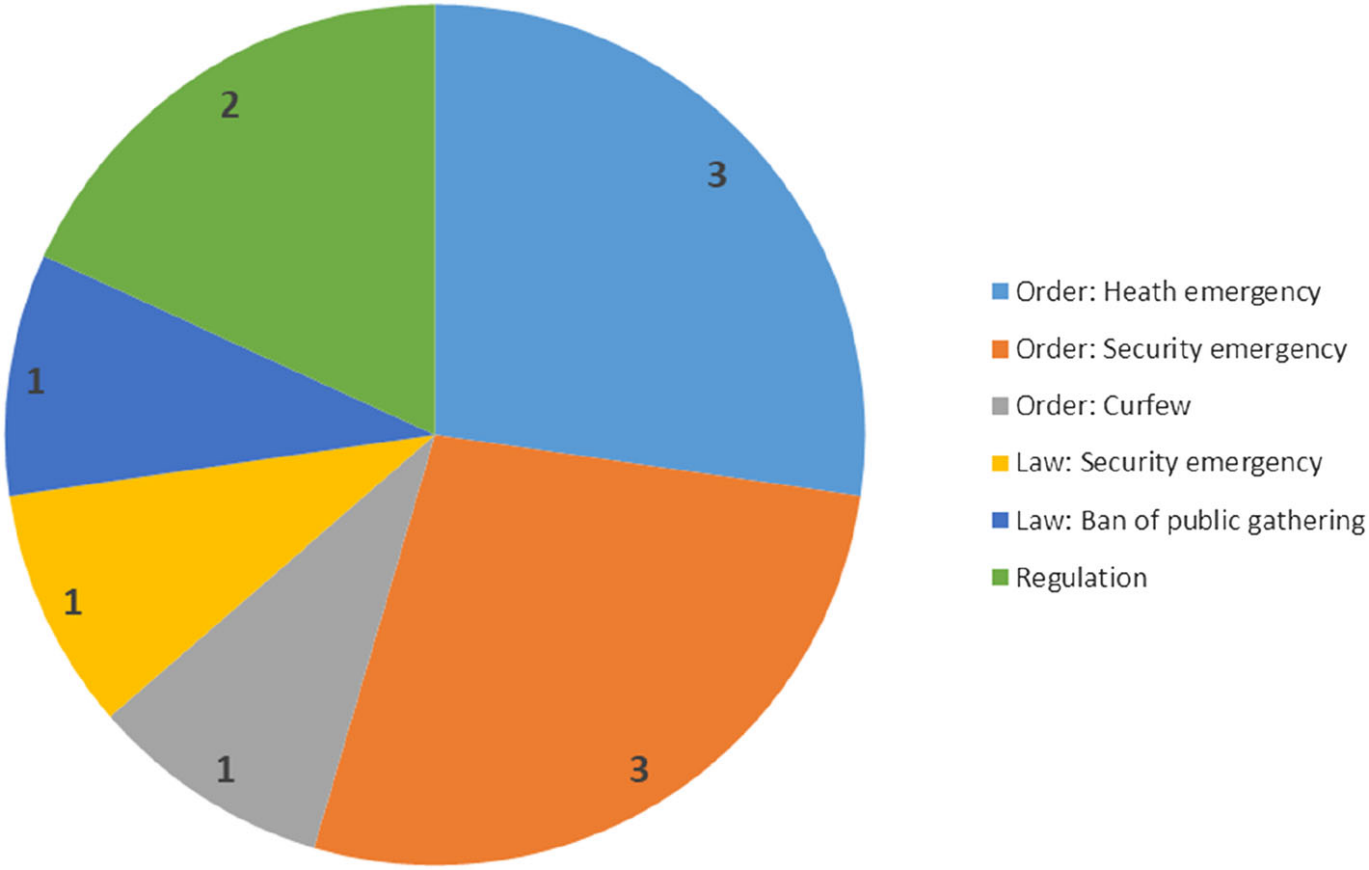
Number of COVID-19-Related Legal Measures Per Type. *Source* Authors’ own compilation, based on ICNL

Orders and decrees passed without parliamentary involvement contribute to executive aggrandizement, undermining the ability of civil societies to legally challenge their governments (Kuehn et. al., 2021). Moreover, both executive and legislative measures that grant wartime-like powers to governments limit rights-based civic space. As governments securitize pandemic responses, they further erode civil society rights and can frame critical civil society as the public enemy to justify repression (Fig. 1).^1^

The Philippines have enforced the most (four) COVID-19-related legal measures. The most problematic is the “Bayanihan to Heal as One Act”, which frames the coronavirus as an existential threat, an “unseen enemy,” and endows president Duterte with wartime-like emergency powers to deploy the armed forces (Hapal, 2021). Passed by Congress, dominated by Duterte’s loyalists, it entails a provision that penalizes what government designates as fake news and can be used by the government to harass political opponents. In fact, the Bureau of Investigation has already brought charges against people criticizing the government’s crisis response online (Castaneda, 2020).

**Fig. 2:**
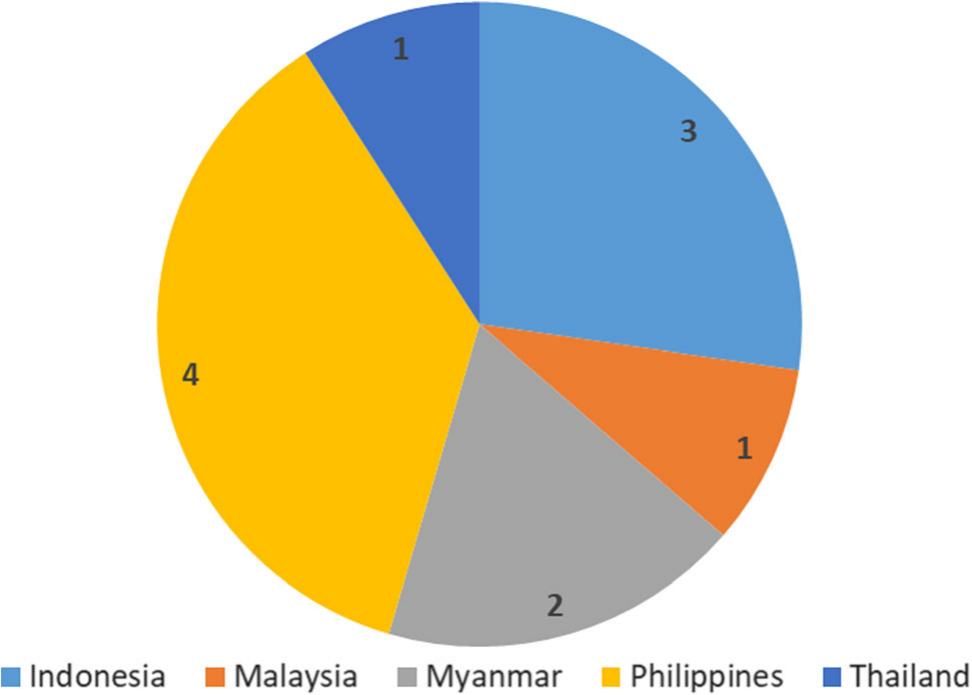
Number of COVID-19-Related Legal Measures Per Country. *Source* Authors’ own compilation, based on ICNL

Second to the Philippines is Indonesia, which imposed three legal measures: one health-related emergency decree and two regulations. However, these mainly focus on restricting movement and social activities for pandemic control and do not frame COVID-19 as a national security threat. The executive is not endowed with wartime-like powers. Accordingly, the authorization of military power in curbing COVID-19’s spread is restrained (Laksmana & Taufika, 2020). Malaysia and Thailand each enacted one legal measure. Malaysia relied on a health emergency-based executive order, while Thailand imposed a security-oriented emergency decree. In Myanmar, the National League for Democracy (NLD)-led government enacted one executive order stipulating regional curfews and one law banning public gatherings that resulted in the arrest of over 500 people (RSF Hub, 2020) (Fig. 2).

However, while such legal measures have curtailed civil society rights, the socioeconomic side effects of COVID-19 have created needs-induced spaces in which civil societies have remained vibrant (Asia Foundation, 2020). According to the World Bank (2021, XII), owing to COVID-19 and related lockdown measures, gross domestic product in 2020 dropped by 2.1 percent in Indonesia, 5.6 percent in Malaysia, 6.1 percent in Thailand, and 9.5 percent in the Philippines, with all these countries experiencing severe rises in inequality. The ASEAN (Association of Southeast Asian Nations) (2020) documented rising unemployment, risks of food insecurity, and drops in education, with marginalized communities, such as informal**-**sector and migrant workers, being disproportionately affected. State responses to the pandemic have often been insufficient and exclusionary, opening space for civil society to mobilize and deliver welfare services. As an NGO representative working in Thailand noted, “the state and the system [are] not going to help us. […] [This] led to a different level of mobilization […] and organizing. So, that’s one opportunity.”^2^

Civil society engagement in COVID-19 and socioeconomic relief has proliferated, enhancing self-organization and invigorating needs-induced civic space. In Indonesia, CBOs and volunteers have delivered social services where state responses have been insufficient and uncoordinated (Jaffrey, 2020). In Gumuk Indah, a self-organized citizens’ Task Force moved from providing health and economic support to veritable self-governance. For instance, Task Force members controlled access to and movement within the village (Sphere Webinar, 2020). A CSO in Malaysia played a similar role in fund-raising and distributing medical supplies to remote public hospitals (Asia Foundation, 2020, 6). In Myanmar, civil society was “responding [to COVID-19] right from the beginning”, a local activist said, implementing hygiene measures and raising awareness where the government failed.^3^ As of mid-2020, the United Nations-led Livelihoods and Food Security Fund estimated that local CSOs implemented more than 80% of its COVID-19 relief measures (Hlaing, 2020). Thailand’s lockdowns also affected tabooed communities, such as sex workers. Service Workers in Groups, whose original mission is providing HIV services, has offered food packs and mental health support to thousands of sex workers excluded from the government’s cash assistance (UNAIDS, 2020).

**Fig. 3:**
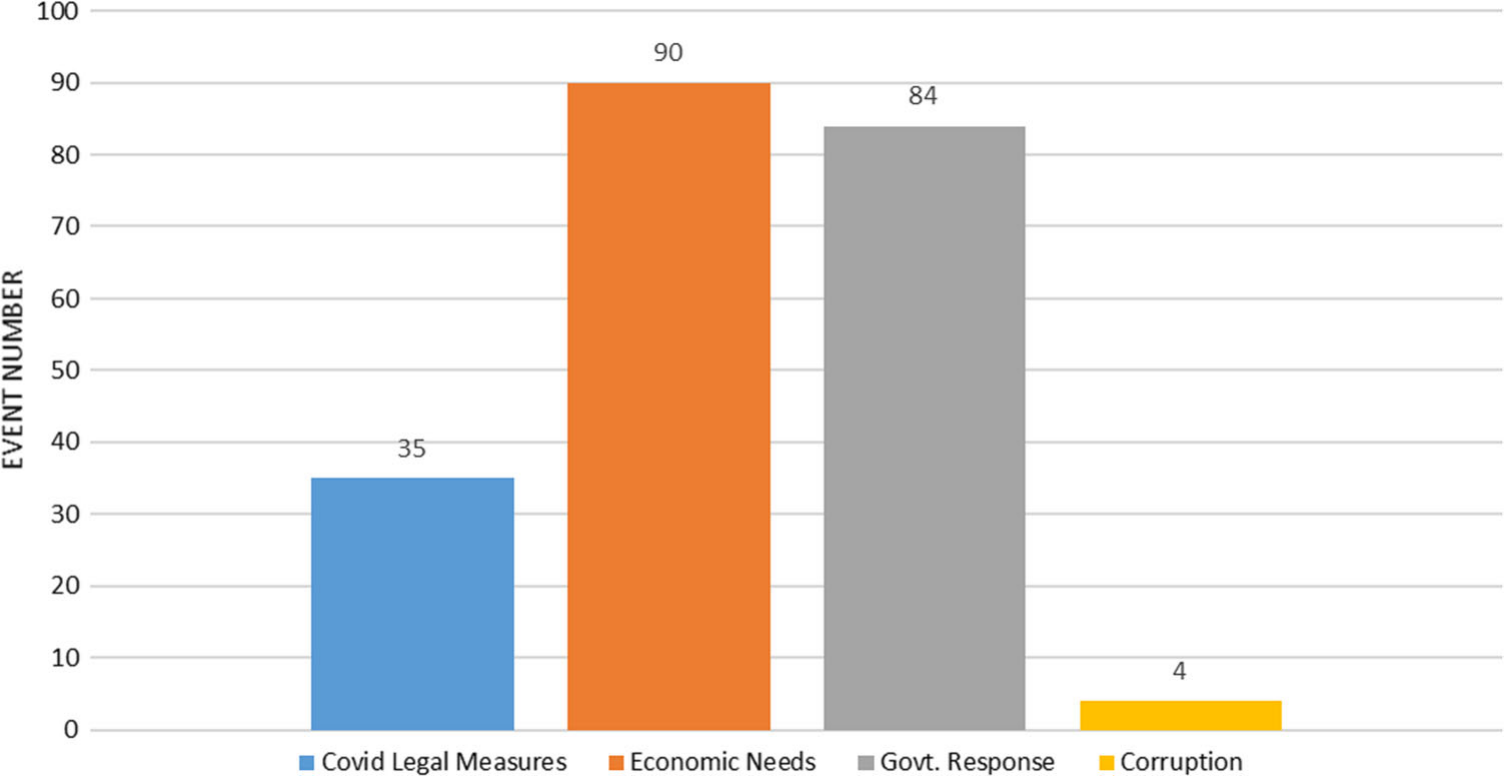
COVID-19-Related Drivers of Protest, April 2020–February 2021. *Source* Authors’ own compilation, based on ACLED

Socioeconomic side effects of lockdowns, in tandem with the inability of incumbent governments to address them, have driven protests across the five countries, indicating that the pandemic’s downsides provide new opportunities for civil society mobilization*.* Out of 2807 protests with more than 100 participants staged in the five countries from April 2020 to February 2021, 213 were directly propelled by COVID-19. The main driving force was economic needs (driver 2) due to movement restrictions and economic shutdowns, often coming in combination with poor government responses to COVID-19 (driver 3).^4^ Informal-sector workers who rely on day-to-day income and do not normally receive welfare subsidies from governments protested to demand unpaid wages from their employers or government assistance. Protesters sometimes criticized harsh lockdowns that hurt poor people or small- and medium-sized businesses. For instance, in August 2020 protests by artists and pub owners in Indonesia demanded easing the existing lockdown to alleviate their financial burdens and business debts. Heavy-handed crackdowns sometimes sparked further civil society action. In the Philippines, some urban poor were arrested in Manila after protesting for livelihood support during the government’s rigorous lockdown (Tomacruz, 2020). Enraged, several CSOs condemned the crackdown, while individual citizens and rights groups expressed their disapproval on Twitter (Billing, 2020) (Fig. 3).

#### Cross-fertilization of Service Delivery and Advocacy Activism

Regarding our second proposition, our data demonstrates the *increasing cross-fertilization of civil society’s service delivery and advocacy activism*. In our roundtable discussion, CSO representatives stressed that there was no “binary”^5^ or “dichotomy”^6^ between advocacy and service delivery. A CSO member from Thailand emphasized that CSOs do not exactly shift from advocacy to service. Rather their activism is “a mix between advocacy and service delivery,” mainly because CSOs’ constituents are in a “vulnerable situation and they do not only need the advocacy […] but they also want the organization[s] that work with them to do some kind of service delivery.^7^”

This cross-fertilization is also evident where originally service-oriented CSO activism has increasingly included efforts to advocate for better welfare policies. In Indonesia, the “SEJAJAR network-of-networks”—which includes 25 national CSO networks and approximately 600 local organizations across 34 Indonesian Provinces—has allowed CSOs to collectively engage the government on welfare issues. It has participated in the government’s National and Provincial Task Forces and dialogued with the ministers in charge of fighting COVID-19 (Pujiono et al., 2020). In Myanmar, the Suu Kyi government in April 2020 established the “National Volunteer Steering Unit” to coordinate all volunteer COVID-19 responses (Mann, 2020). Some CSO representatives criticized this and similar moves as government attempts to control civil society, while failing to adequately support civil society-based initiatives.^8^ Accordingly, many continued to work independently, while linking service delivery to human rights advocacy. In May 2020, over 200 CSOs issued a statement urging the government to respect democracy, human rights, and social justice in its crisis response (TNI, 2020).

In addition, many advocacy CSOs have increasingly moved into service delivery, while simultaneously retaining their political advocacy.^9^ In the Philippines, Active Vista Center and other human rights groups have included service delivery into their agendas, while challenging the Duterte government’s militaristic COVID-19 response and advocating for equal access to public services (Savage, 2020). Similarly, pro-democracy activists in Thailand, in parallel with their 2020 anti-regime protests, provided relief to slum dwellers and other marginalized communities (Chuipracha, 2020). The CSO We Fair, which emerged from these anti-regime protests, explicitly highlights the linkage between service delivery and civil rights. Specifically, the group has advocated for better welfare policies, including increased wages, pension, and affordable education, to address the nexus between economic inequality and autocracy (Matichon, 2021). In Myanmar, ethnic minority activists have moved from human rights campaigns to health advocacy and service provision (Quadrini, 2020). Based on interviews with 47 CSOs from Southeast Asia, the Asia Foundation (2020, 2) concludes that engagement in COVID-19 relief “allows CSOs to simultaneously engage in and contest government policies and policymaking, and to serve as two-way conduits between communities and governments”.

#### Sustained Civic Space

Supporting our third proposition, the findings derived from our roundtable, interviews, and gray literature show that *the cross-fertilization of service and advocacy activism works to sustain civic space, including by politicizing some formerly apolitical spaces in welfare provision*. Oftentimes, this has involved coordination and critical engagement with government agencies. In Malaysia, the government’s Movement Control Order initially barred NGOs from helping migrant and refugee communities, directing them to channel all aid through the state welfare department and the paramilitary RELA Corps. NGOs ignored the instructions, however, while advocating for the government to work with civil society. Realizing it relied on the NGOs’ resources and capabilities in reaching marginalized communities, the government gave in, issued new NGO guidelines, and increased its collaboration with CSOs (Ambrose, 2020; Chen, 2020). In January 2021, 46 health experts—including representatives of medical associations—published an open letter urging the government to adopt a “whole-of-society approach,” to expand “tripartite government, private sector and NGO partnership[s]” and to set up an independent “COVID-19 Task Force” (Malaysiakini, 2021). The prime minister publicly embraced the whole-of-society approach and encouraged the health experts to nominate members for a “Health and Scientific COVID-19 Advisory Group” that would counsel the government (PMO Malaysia, 2021). Similarly, in Indonesia, the Home Ministry in October 2020 issued Circular Letter 440/5538/SJ that encourages the involvement of CSOs in COVID-19 relief, reflecting the government’s realization of CSOs’ indispensable contributions to the national crisis response. Importantly, the circular also introduces a new procurement scheme that enables the active participation of CSOs in public procurement (KSI, 2020), a measure that CSOs had already advocated before the pandemic.

**Fig. 4:**
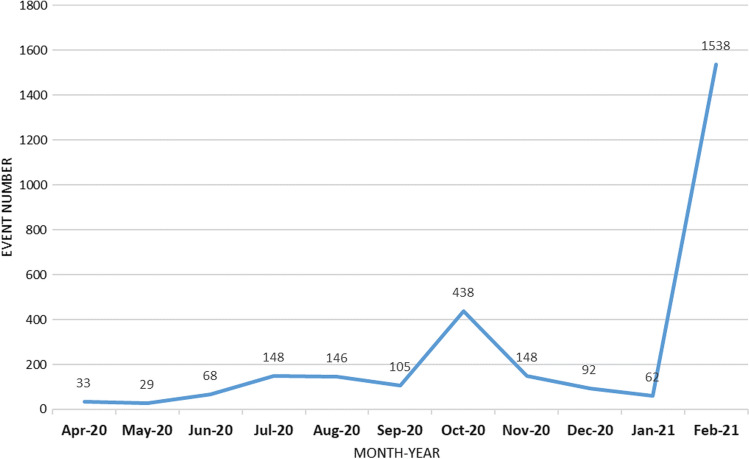
Protest Events per Month, April 2020–February 2021. *Source* Authors’ own compilation, based on ACLED

A more quantitative look at advocacy activism expressed through street protests likewise confirms that civic space has often been sustained. In the five Southeast Asian countries studied, mass demonstrations not directly related to COVID**-**19 have persisted and proliferated despite restrictions on civil society rights and, at times, massive repression. From April 2020 to February 2021, 2594 out of the 2807 major protest events we coded were driven by political reasons and not directly related to COVID-19*.* Such episodes were infrequent from April to June 2020, but the number more than doubled thereafter, from 2226 events in the period from April 2019 to March 2020 to 5459 events in the period from April 2020 to March 2021 (Fig. 4).^10^

**Fig. 5:**
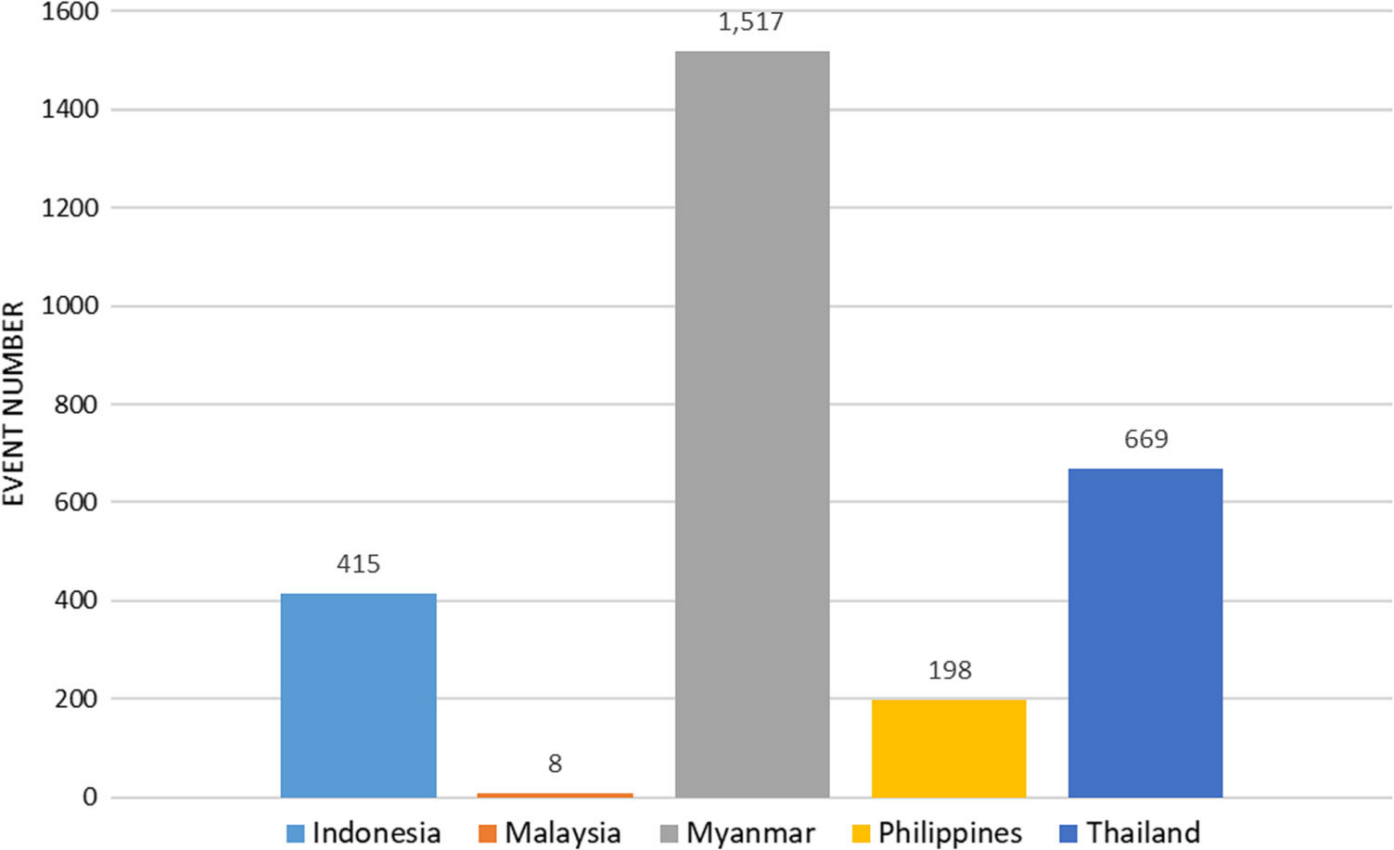
Protest Events per Country, April 2020–February 2021. *Source* Authors’ own compilation, based on ACLED

Thailand and Myanmar saw most protests, with the third and fourth ranks being held by Indonesia and the Philippines, respectively*.* In both countries, CSOs and individual citizens defied military rule despite massive risks for their safety. In Myanmar, the military ultimately quelled large-scale protests with utmost brutality, but flash mob protests have persisted. In Thailand, where COVID-19 deepened economic disparities, the pandemic has catalyzed mobilization against the competitive autocratic regime framed by protesters as neglecting citizens’ socioeconomic plight*.* Oftentimes, legal threats and arrests of key protest activists even further motivated the masses to join the resistance (Sombatpoonsiri, 2021). In Indonesia, citizens protested against the draft omnibus laws that would relax environmental and wage standards. The police used force to disperse the protests, aggravating them into riots (Warganegara, 2020) (Fig. 5).

In addition, discussions among our roundtable participants suggest that CSOs that have provided services have sometimes politicized formerly apolitical spaces in welfare delivery by framing pandemic-related popular needs as representations of long standing grievances caused by neoliberal development models and advocating for more inclusive models of state-society relations. Philippine CSO activists who came from a leftist political tradition but have increasingly provided services and advocated successive governments for more inclusive and participatory welfare policies stressed the potential of “disruption smart” CSOs to actively use COVID-19 to advance political reforms:

[D]isruption smart activists can take advantage of these disruptions [like COVID-19]. […] A skillful activist […] can insert really progressive, inclusive policies in the frame […] during the crisis. So [...] that’s one opportunity and [...] civil society has to learn how to enter into that [needs-induced] space skillfully […] to use the disequilibrium to advance a progressive agenda.^11^

A CSO activist working in Myanmar argued that “service delivery in itself can be a political act depending on who you’re providing services to.”

Our roundtable participants further emphasized that COVID-19 relief had allowed them to strengthen their ties with local communities and to engage the latter in building state-society relations that combine the establishment of community-based service systems with pressure for strong welfare states (see footnote 11). A CSO representative from Thailand noted that in the medium-term advocacy campaigns needed to focus on the welfare system (see footnote 11). Another activist emphasized the need for new “society-state systemic partnerships”, arguing that “the state must […] provide the systems […] needed to address inequality” and that CSOs must not enter “noncritical partnerships” with governments (see footnote 11).

## Conclusion

We have shown that during COVID-19 civic space has often been sustained in Indonesia, Malaysia, Myanmar, Thailand, and the Philippines. In all five countries, civic space has not been configured solely by legal restrictions, but by the interplay of these restrictions, needs-induced space, and civil society activism. Specifically, COVID-19-related legal restrictions have intertwined with needs-induced space to create new opportunities for civil society activism. Within these new opportunity structures, the cross-fertilization between service delivery and advocacy activism has allowed advocacy CSOs to continue thriving in the context of shrinking rights-based space, prompted service CSOs to take more assertive stances toward their governments, and politicized formerly apolitical spaces in welfare provision. These interlocking mechanisms have worked to sustain civic space.

Our findings contribute to the nascent research on civil society pushback against shrinking space. Specifically, they show that beyond pushing back against NGO laws, CSOs can also preserve civic space by leveraging their contributions in service delivery to bring governments to tolerate more political civil society activism. This insight also speaks to the broader literature on civil society, which often associates service delivery with apolitical CSOs accommodative to restrictive regimes. Contrasting this conventional wisdom, our findings show that CSOs may use the enhanced engagement in service delivery as a deliberate means to work against civic space restrictions. Future research should investigate to what extent our three determinants of civic space and mechanisms of how they interact, which we undergirded with empirical evidence from five flawed democracies and competitive autocracies in Southeast Asia, are also applicable to other world regions and closed autocracies. An additional possibility is to explore how the interlinkage of the three determinants of civic space is impacted by other factors, such as historical legacies (Rakner, 2021), the strength and nature of opposition parties (Bernhard et al., 2020), enduring civil-society-elite relations, and existing patterns of cooptation and clientelistic networks (Lorch, 2021).

In terms of practical implications, our findings remind Southeast Asian CSOs of the necessity to nurture holistic forms of activism in the face of shrinking rights-based space. This is especially important because the pandemic has exposed the weaknesses of existing models of socioeconomic development, opening up possibilities for civil society to advocate for more inclusive state-society relations. To achieve this, civil society activists should build broad-based coalitions that further deepen the various forms of cross-fertilization of service and advocacy activism that have emerged during the pandemic.

Rather than guiding pushback against civic space restrictions, INGOs and international development agencies should support the “localization” of civil society-based resilience mechanisms to protect civic spaces (Sriskandarajah, 2017). In this way, they cannot only help preserve spaces for CSO operations, but also strengthen more autonomous and democratic forms of civil society activism.

## Data Availability

This article is drawn from datasets that are constitutive parts of our ongoing research project on civic space and COVID-19 (funding source identified above). The datasets are yet to be finalized and may be published via SowiDataNet|datatorium data repository that hosts data originating from research undertaken by instiutes under the Leibniz-Institut für Sozialwissenschaften.

## Footnotes

1 Between April 2020 and February 2021, out of the 12 types of legal measures we coded only eight were enacted in the five countries. We found no orders or decrees banning public gathering or restricting information. Similarly, governments enacted no laws that stipulate curfews or regulate NGO activities. Thailand introduced a new NGO law in April 2021, which has, however, not been passed by parliament as yet.

2 Online CSO Roundtable, 1 June 2021.

3 Remote interview, Myanmar CSO representative, 15 July 2020.

4 There was no reported protest driven by sexual violence. We omit this driver in the graph accordingly.

5 Online CSO roundtable, 1 June 2021.

6 Online CSO roundtable, 1 June 2021.

7 Online CSO roundtable, 1 June 2021.

8 Remote interview, Myanmar CSO representative, 15 July 2020; email conversation, Myanmar CSO leader, 13 July 2020.

9 Online CSO roundtable, 1 June 2021.

10 Unlike the 2807 major protests with more than 100 participants we coded to identify their drivers, these figures are extracted directly from the ACLED database. They include protests with less than 100 participants.

11 Online CSO roundtable, 1 June 2021.
